# Evaluation of urban ecological livability and obstacle factor diagnosis from a synergistic perspective: A case study of Zhangjiajie City, China

**DOI:** 10.1371/journal.pone.0313267

**Published:** 2024-11-15

**Authors:** Mingrong Deng, Shenhai Zhu, Bihu He, Heli Zhang

**Affiliations:** 1 School of Civil Engineering and Architecture, Jishou University, Zhangjiajie, China; 2 Yongxing County Natural Resources Bureau, Chenzhou, China; Shahid Beheshti University, ISLAMIC REPUBLIC OF IRAN

## Abstract

The construction of ecological livability plays a crucial role in achieving ecological civilization. During economic development, conflicts between urban populations, resources, and the environment have become increasingly prominent. Although China’s efforts in building ecological livability have made certain achievements, they are still in the early stages, making research in this area very necessary. This study aims to establish an indicator system for the level of ecological livability construction from four aspects: ecology, society, residents’ living conditions, and economy. Based on panel data from 2008 to 2021, the entropy-TOPSIS model was used to evaluate the level of ecological livability construction in Zhangjiajie City. Additionally, the coupling coordination degree model was employed to study the correlation and coordination among different indicators within the system. Furthermore, the obstacle degree model was introduced to explore the main factors affecting the improvement of ecological livability construction levels. The results show that, over the fourteen-year period, the proximity of Zhangjiajie City’s ecological livability construction level decreased from 0.2712 in 2008 to 0.2547 in 2010, then rose to 0.7686 in 2021, reaching a medium coordination level, with the constraining effects among the ecological livability systems weakening. The average wage of employees, the proportion of social security and employment expenditure to fiscal expenditure, the per capita total postal and telecommunications business volume, the per capita number of public library collections, and the proportion of education expenditure to fiscal expenditure have become the main influencing factors of Zhangjiajie City’s ecological livability construction level.

## Introduction

In the process of urban development, the contradictions among urban population, resources, and the environment have become increasingly prominent [1, 2]. Humans often overlook the balance between economic development and environmental protection, leading to a series of "urban diseases", such as traffic congestion, housing shortages, environmental pollution, and regional development imbalances, seriously disrupting the ecological environment of cities [3–5]. The imbalance between the socio-economic benefits and ecological environmental benefits brought about by urbanization has become a widespread global phenomenon [6]. As early as 1961, the World Health Organization defined "safety, health, convenience, and comfort" as the four basic concepts of the living environment [7]. In order to address these urban crises and achieve sustainable development in human habitats, various concepts of urban development models have been proposed.

Since the late 20th century, urban planners have put forth various visions to improve the quality of life for urban residents [8, 9]. For instance, Ebenezer Howard proposed the concept of the "garden city" focusing on addressing issues such as urban environmental pollution, population expansion, housing comfort, transportation convenience, and environmental health [10]. As people’s demands for urban functionalities continue to evolve, concepts like satellite cities, landscape cities, and low-carbon cities have emerged [11–14]. Although there isn’t complete consensus on urban visions, these ideas have sparked a new concept in urban development known as livable cities. Livable cities encompass various aspects, including social, economic, and environmental factors [15–17]. Typically applied in urban planning, it involves a multi-faceted indicator system to measure the quality of community life. This system considers factors such as the natural environment, economic development, social stability and equity, educational opportunities, culture, and opportunities for entertainment and leisure [18–20]. The goal of a livable city is to enhance various aspects of urban life, making it easier for people to reside and work within it [21, 22].

Simultaneously, the concept of ecologically livable cities places a greater emphasis on considering the vulnerability of regional ecosystems and focuses on the quality of the surrounding environment for local residents, highlighting the significance of ecology in the planning and construction of livable cities [23]. In the 19th National Congress report in China, "ecological livability" is identified as an important aspect, underscoring the philosophy that "green waters and lush mountains are invaluable assets" [23]. Since then, ecologically livable cities have become a focal point of research among Chinese scholars. It is worth noting that the concepts of livable cities and ecologically livable cities currently lack clear definitions, but both are proposed concepts aimed at achieving sustainable urban development. Livable cities emphasize a people-centric approach, while ecologically livable cities place a greater emphasis on the importance of ecological factors in urban construction [24–26].

In the research on ecologically livable cities, primary considerations include climate conditions, ecological environmental changes and factors such as local residents’ social welfare and income disparity [27–30]. Previous studies have focused on aspects such as urban environmental quality, living conditions, and green evaluations [31, 32]. When examining the impact of economic development on livable cities, scholars have proposed two different viewpoints. Some scholars believe that economic development enhances a city’s ecological livability level, as urban scale and quality gradually improve with economic growth [18, 33]. The opposing view argues that economic development has a negative impact on urban ecological livability, suggesting that urban expansion can reduce residents’ satisfaction with transportation and housing [34, 35]. Additionally, some research indicates that economic development is not the sole determinant of a city’s ecological livability level, emphasizing that the convenience of urban life is also crucial to the construction of an ecologically livable city, as it reflects residents’ perceptions and the quality of public facilities such as shopping, education, healthcare, culture, and entertainment [36]. Jiang et al., proposed a conceptual model incorporating various economic, social, and environmental factors, establishing a multi-factor indicator system for the analysis of Fujian Province [7]. Additionally, some scholars argue that people’s perception of ecologically livability is also an important influencing factor. Fan et al., considering citizen, ecological, and urban dimensions, developed an evaluation indicator system for urban ecological livability and conducted an empirical study on the ecological livability of the Guangdong-Hong Kong-Macao Greater Bay Area [24]. As research deepens, the role of technology and talent in urban development and achieving ecological livability has been recognized [37, 38]. Therefore, indicators related to technology, such as the number of research scientists and internal expenditures on research and development funds, have been introduced [3]. In terms of evaluation methods, due to the close connection between ecological livability and human life, scholars tend to adopt a combined quantitative and qualitative approach. For instance, Saeideh et al., utilized on-site machine-measured microclimatic data and combined it with a questionnaire survey to have citizens vote on thermal comfort, providing both qualitative and quantitative data for subjective and objective assessments [39]. At the same time, scholars actively explore more scientific methods, such as remote sensing, ArcGIS spatial analysis, coupling coordination models, and the Technique for Order Preference by Similarity to an Ideal Solution (TOPSIS) method, to calculate the ecological livability level of the study area [40–43]. Furthermore, they apply system dynamics models to identify the mutual relationships between influencing factors [19, 44].

To scientifically measure the ecological livability construction level of Zhangjiajie City, analyze the interactions among its various systems, and identify the main obstacles hindering its ecological livability construction level, this study proposes corresponding countermeasures and suggestions. These measures aim to achieve the coordinated development of Zhangjiajie City’s ecological livability construction level among people, nature, society, and the economy. This study employs the entropy weight method to modify the evaluation values of positive and negative ideal solutions in the TOPSIS model. The traditional TOPSIS method relies mainly on subjective judgments from experts for weight determination, posing a risk of results deviating from reality. The entropy weight method, being more objective, is utilized to construct an entropy-TOPSIS model for evaluating the performance of soil and water conservation projects. Previous research has focused on measuring the level of ecological livability construction, with less consideration for the adaptability between systems. Coupling theory can be applied to describe the degree of interaction between multiple systems. Utilizing the coupling coordination model can effectively observe the coordination status of various systems. Combining the obstacle diagnosis model provides a better explanation for the shortcomings in the improvement of ecological livability construction levels. This approach offers targeted scientific recommendations.

## Materials and methods

### Study area

This study selected Zhangjiajie City, Hunan Province, China, as the research area. Zhangjiajie City is located in the northwest of Hunan, in the core area of the Wuling Mountain region, between 28°52′ and 29°48′ latitude and 109°40′ to 111°20′ longitude. The city experiences a subtropical monsoon climate with distinct seasons, abundant rainfall, ample sunshine, and a beautiful urban environment, which contribute to living and habitation. Additionally, Zhangjiajie is renowned as one of China’s most important tourist cities, serving as a key national ecological functional area and a vital ecological barrier in the middle and lower reaches of the Yangtze River. In the 14th Five-Year Plan for Zhangjiajie City, it is proposed to build a National Ecological Civilization Pilot Zone (NECPZ), a beautiful symbol of China, and a pleasant and livable, happy and beautiful home for both work and residence. The plan continues the people-oriented urban development philosophy, focusing on improving the quality of the ecological environment, addressing deficiencies in social infrastructure, and enhancing the level of public services. The goal is to create a harmonious, livable, and vibrant city, improve the living environment, promote sustainable economic and social development, and strive to provide the residents with a more ecological and livable city that brings a greater sense of achievement, happiness, and security.

### Data sources

The original data required for the livability comprehensive evaluation index system, including ecological development level, public services and security, residential life and cultural environment, and economic development level, were obtained from *Statistical Yearbook of Hunan Province* and *Zhangjiajie City Statistical Yearbook*, most of which are the original data of the statistical yearbook, and the other the index data are calculated by using the original data, which can be found at Supporting Information (S1 Table).

### Construction of the indicator system

Drawing on the livability assessment frameworks from various countries in recent years, the regional ecological livability system is subdivided into four subsystems: ecological development level, public services and security, residential life and cultural environment, and economic development level. On the basis of data availability, indicators representing the comprehensive level of regional livability were extracted to construct the livability evaluation index for Zhangjiajie City (Table 1).

**Table 1:** The evaluation index system for the ecological livability construction level in Zhangjiajie.

Target Layer	Primary indicator system	Indicator content	Index type	Weights
Ecological livability construction level (A)	(B1) Ecological development level	(C11) Energy Consumption per Unit of GDP (Ton of standard coal/10,000 RMB)	-	0.0290
		(C12) Electricity consumption per unit of GDP (kW·h//10,000 RMB)	-	0.0254
		(C13) Water consumption per unit of GDP (m^3^/10,000 RMB)	-	0.0288
		(C14) Green coverage rate of built-up area (%)	+	0.0315
		(C15) rate of good air quality (%)	+	0.0318
		(C16) Forest coverage rate (%)	+	0.0443
		(C17) Treatment rate of domestic sewage (%)	+	0.0279
		(C18) Hazzard-free treatment rate of domestic garbage	+	0.0382
	(B2) Public services and security	(C21) Natural gas coverage rate (%)	+	0.0237
		(C22) Highway miles per 10,000 people (km/10,000 people)	+	0.0217
		(C23) Number of doctors per 10,000 people (ten thousand person)	+	0.0373
		(C24) Number of hospital beds per 10,000 people (ten thousand person)	+	0.0386
		(C25) Urban registered unemployment rate (%)	-	0.0247
		(C26) The ratio of disposable income to house price of urban residents	-	0.0200
		(C27) Average wage of in-service employees (RMB)	+	0.0394
		(C28) Proportion of social security and employment to fiscal expenditure (%)	+	0.0306
		(C29) Per capita postal and telecommunications business volume (RMB/people)	+	0.0392
	(B3) Residential life and cultural environment	(C31) Population density (per people/km^2^)	-	0.0216
		(C32) Natural population growth rate (%)	-	0.0236
		(C33) Per capita savings deposit balance	+	0.0299
		(C34) Number of household cars per hundred households	+	0.035
		(C35) University students per 10,000 people (ten thousand people)	+	0.0265
		(C36) Basic education student-teacher ratio	+	0.0239
		(C37) Per capita public library book collection	+	0.0391
		(C38) Proportion of education expenditure to fiscal expenditure (%)	+	0.0314
	(B4) Economic development level	(C41) Per capita GDP (RMB)	+	0.0338
		(C42) Proportion of tertiary industry to GDP (%)	+	0.0166
		(C43) Per capita retail sales of social consumer goods (RMB)	+	0.0340
		(C44) Per capita fixed asset investment (RMB)	+	0.0262
		(C45) The urbanization rate (%)	+	0.0331
		(C46) Urban-rural income ratio	+	0.0375
		(C47) Proportion of added value of high-tech industries to GDP (%)	+	0.0281
		(C48) Labor productivity (RMB/people)	+	0.0277

In terms of ecological development level: The ecological environment is a key component of ecological livability, and the ecological livability of a region plays an increasingly important role in influencing environmental quality. The ecological development level reflects the natural environment and environmental hygiene conditions of the region. A comfortable environment is positively correlated with residents’ sense of well-being, while the intensification of environmental pollution can diminish residents’ subjective sense of happiness [45]. This work employed energy consumption [46], per Unit of GDP, electricity consumption per unit of GDP, water consumption per unit of GDP [47], green coverage rate of built-up area [45], rate of good air quality [3, 45], forest coverage rate [3], treatment rate of domestic sewage and Hazzard-free treatment rate of domestic garbage [42, 48] to evaluate ecological development level.

In terms of public services and security: Public services and security contribute to enhancing residents’ overall satisfaction. This aspect encompasses two levels—social services and social security, which constitute the "soft environment" influencing the livability of urban areas. It directly reflects residents’ satisfaction with the basic social functions of the city. Strengthening the social security and service systems is a key aspect of urban development and social progress [47]. Therefore, the public services and security is represented by natural gas coverage rate[47], highway miles per 10,000 people [49], number of doctors per 10,000 people [42, 45], number of hospital beds per 10,000 people [50], urban registered unemployment rate [46, 51], the ratio of disposable income to house price of urban residents [11, 48], average wage of in-service employee [48], proportion of social security and employment to fiscal expenditure [46], per capita postal and telecommunications business volume [52].

In terms of the residential life and cultural environment: The construction of the human living environment in ecological livability should focus on the daily activities of residents. Therefore, when selecting indicators, it is essential to fully consider resident-related factors, with particular attention to population factors. The residential life aspect primarily reflects the impact of population size and growth rate, population quality, and income security on residents’ lives. The cultural environment mainly examines the scale of construction of urban information, education, and public activity venues and facilities. It reflects residents’ satisfaction with cultural life, which can enhance their overall satisfaction with life to some extent, and indicates urban residents’ perception of and accessibility to the public environment [36]. The residential life and cultural environment is represented by population density[24], natural population growth rate [52], per capita savings deposit balance [53], number of household cars per hundred households, university students per 10,000 people [45, 52], basic education student-teacher ratio [24], per capita public library book collection [46, 54] and proportion of education expenditure to fiscal expenditure [24].

In terms of economic development level: Healthy and sustainable economic development is an essential material foundation for improving the level of ecological livability. Economic conditions are the basic material prerequisites for achieving a better life for residents, providing the foundation for enjoying a better ecological environment, public services, and living conveniences, which in turn enhances the overall standard of living. Similarly, the economic development level includes per capita GDP [3, 55], proportion of tertiary industry to GDP [55], per capita retail sales of social consumer goods [3], per capita fixed asset investment [47], urbanization rate [46, 48], urban-rural income ratio [48], proportion of added value of high-tech industries to GDP [48] and labor productivity [3].

### Study method

Entropy weight method. This study employs an objective analysis method, namely the entropy weight method, to determine the weights of the indicators. The entropy weight method is suitable for comprehensive evaluations involving multiple indicators to determine their weights. In the process of solving indicator weights, there are generally two approaches: subjective and objective weighting methods [41, 56]. Typically, subjective weight determination methods include the Analytic Hierarchy Process (AHP). However, a major issue with this method is its inability to effectively address inappropriate subjective weight allocations, which can lead to a failure in fully reflecting the information of each indicator, potentially causing biases in the assessment results [57]. In contrast, the entropy weight method is an objective approach that uses information entropy to determine weights. The smaller the entropy value of an indicator, the more information it contains, and the higher its weight. Conversely, the larger the entropy value, the less information the indicator contains, and the lower its weight [58]. This method determines weights based on the relationships among different indicators, ensuring that the evaluation results are not influenced by personal subjective judgments. It involves various multivariate analysis techniques and offers high scientific reference value [59], making it widely applied in the evaluation of socio-economic development [60]. This study utilizes the entropy weight method to calculate the weights of indicators for ecological livability levels, with the specific calculation steps outlined below.

Step1: Standardized data processing:

$$Positive\;indicator:\;x{'}_{ij}=\frac{x_{ij}-\min(x_{ij})}{\max(x_{ij})-\min(x_{ij})}$$ (1)

$$Negative\;indicator:\;x{'}_{ij}=\frac{\max(x_{ij})-x_{ij}}{\max(x_{ij})-\min(x_{ij})}$$ (2)

Where the “range normalization” method is adopted, where *i* = 1, 2, …, *n* and *j* = 1, 2, …, *m*, x’_ij_ is the normalized value of x_ij_; max(x_ij_) is the maximum value of the *j*-th indicator over *i* years, and min(x_ij​_) is the minimum value of the *j*-th indicator over *i* years.

Step2: Calculate the proportion of the *j*-th indicator value *y*_*ij*_.


$$y_{ij}=\frac{x{'}_{ij}}{\sum_{i=1}^{m}x{'}_{ij}}$$ (3)

Step3: Calculate the entropy of the indicators e_j_:

$$e_{j}=-\frac{1}{\ln\;n}\sum_{i=1}^{n}y_{ij}\ln y_{ij}$$ (4)

Step4: Calculate the coefficient of variation for the *j* indicator d_j_:

$$d_{j}=1-e_{j}$$ (5)

Where *e*_*j*_ represents the calculation results of indicator *j*.

Step5: Calculate the weights of the indicators u_j_:

$$u_{j}=\frac{d_{j}}{1-\sum_{j=1}^{n}d_{j}}$$ (6)

### TOPSIS model

The TOPSIS is a technique used to rank evaluation objects based on their proximity to an idealized target. The core idea of this method is to determine the positive ideal solution and negative ideal solution for each indicator. Then, by calculating the weighted Euclidean distance between each option and the PIS or NIS, a comprehensive evaluation is conducted to determine the degree of proximity to the optimal solution [61]. The positive ideal solution refers to an imagined solution where all attribute values are at their optimal levels, while the negative ideal solution refers to an imagined solution where all attribute values are at their worst levels. If an evaluation object is closest to the positive ideal solution and furthest from the negative ideal solution, it is considered the optimal solution; conversely, if it is furthest from the positive ideal solution and closest to the negative ideal solution, it is considered the least optimal solution. The TOPSIS model balances the volatility of indicator data while maintaining the trend and distribution of the data, thereby improving the scientific rigor and accuracy of the evaluation [56]. The main calculation steps are as follows:

Step1: Construct the weighted decision matrix. In the preprocessing of the original data, obtain the decision matrix $X={\left[x{'}_{ij}\right]}_{mn}$ through min-max standardization. Multiply each row of the standardized matrix $X={\left[x{'}_{ij}\right]}_{mn}$ by u_j_ to obtain the weighted decision matrix $S={\left[s_{ij}\right]}_{mn}$.

Step2: Calculate the positive (Q^+^) and negative ideal solutions (Q^-^). The calculation formulas are as follows:

$$Q^{+}=\{\max\;s_{ij}|j=1,2,\cdots ,n\}=\{{Q_{1}}^{+},{Q_{2}}^{+},\cdots ,{Q_{n}}^{+}\}$$ (7)

$$Q^{-}=\{\min\;s_{ij}|j=1,2,\cdots ,n\}=\{{Q_{1}}^{-},{Q_{2}}^{-},\cdots ,{Q_{n}}^{-}\}$$ (8)

Step3: Calculate the distance of each object to the optimal solution (L_j_^+^) and worst solution (L_j_^-^). The calculation formulas are as follows:

$$L_{j}^{+}=\sqrt{\sum_{j=1}^{n}{\left(Q_{j}^{+}-s_{ij}\right)}^{2}}$$ (9)

$$L_{j}^{-}=\sqrt{\sum_{j=1}^{n}{\left(Q_{j}^{-}-s_{ij}\right)}^{2}}$$ (10)

Step4: Calculate the approach degree. The approach degree represents the proximity of each objective to the optimal level, with a value range of [0, 1]. A higher approach degree indicates that the objective result is closer to the optimal solution. The calculation formula is as follows [62]:

$$F_{j}=\frac{{L^{-}}_{j}}{{L^{+}}_{j}+{L^{-}}_{J}}$$ (11)

Where Fj represents the evaluation results of relative proximity, and its value ranges from 0 to 1.

### Coupling coordination degree model

The coupling theory can be employed to describe the degree of interaction among multiple systems. Currently, commonly used formulas for coupling degree models are as follows:

$$C={\left[\frac{\prod_{i=1}^{n}u_{i}}{\left(\frac{1}{n}\sum_{i=1}^{n}u_{i}\right)}\right]}^{\frac{1}{n}}$$ (12)

$$T=\sum_{i=1}^{n}a_{i}u_{i},\sum_{i=1}^{n}a_{i}=1$$ (13)

$$D=\sqrt{C\times T}$$ (14)

Where n is the number of systems, U_i_ represents the comprehensive development index of each system, distributed within the interval [0,1]. Therefore, the coupling degree value C ranges between 0 and 1, with higher values indicating lower disparity among systems and a higher degree of coupling. T is the comprehensive coordination development index, and a_i_ is the weight of i systems, and D is the coupling coordination degree. The classification criteria of coupling degree and coupling coordination degree can be founded in Tables 2 and 3.

**Table 2:** The criteria for classifying coupling degree.

Coupling degree	Coupling level	Coupling degree	Coupling level
(0,0.1]	Extremely decoupling	(0.5,0.6]	Weakly coupling
(0.1,0.2]	Severely decoupling	(0.6,0.7]	Primary coupling
(0.2,0.3]	Moderately decoupling	(0.7,0.8]	Intermediate coupling
(0.3,0.4]	Slightly decoupling	(0.8,0.9]	Good coupling
(0.4,0.5]	Marginally decoupling	(0.9,1.0]	Excellent coupling

**Table 3:** The criteria for classifying coupling coordination degree.

Coupling coordination	Coupling coordination level	Coupling coordination	Coupling coordination level
(0,0.1]	Extremely imbalanced	(0.5,0.6]	Marginally coordinated
(0.1,0.2]	Severely imbalanced	(0.6,0.7]	Primary coordinated
(0.2,0.3]	Moderately imbalanced	(0.7,0.8]	Intermediate coordinated
(0.3,0.4]	Slightly imbalanced	(0.8,0.9]	Well-coordinated
(0.4,0.5]	On the verge of imbalance	(0.9,1.0]	High-quality Coordinated

### The diagnosis model of obstacle factor

Analysis of the main obstacle factors affecting the ecological livability construction level of Zhangjiajie City can provide targeted development recommendations for enhancing the ecological livability construction level [63]. The specific steps involve introducing three basic variables: factor contribution T_j_, index skewness I_j_, and influence degree G_j_. Generally, the weights u_j_ of each indicator are used to represent the contribution of a specific indicator to the overall goal (the ecological livability construction level of Zhangjiajie City). The index skewness I_j_ is expressed as the difference between 1 and the standard value when determining the weight of each indicator, representing the distance of each indicator’s actual value from the optimal target value. The influence degree indicates the magnitude of each indicator’s impact on the ecological livability construction level of Zhangjiajie City and is calculated using the following formula [64]:

$$G_{j}=\frac{I_{j}\cdot T_{j}}{\sum_{j=1}^{n}I_{j}\cdot T_{j}}\times 100\%$$ (15)

## Results and discussion

### Ecological livability construction level analysis

**Fig 1:**
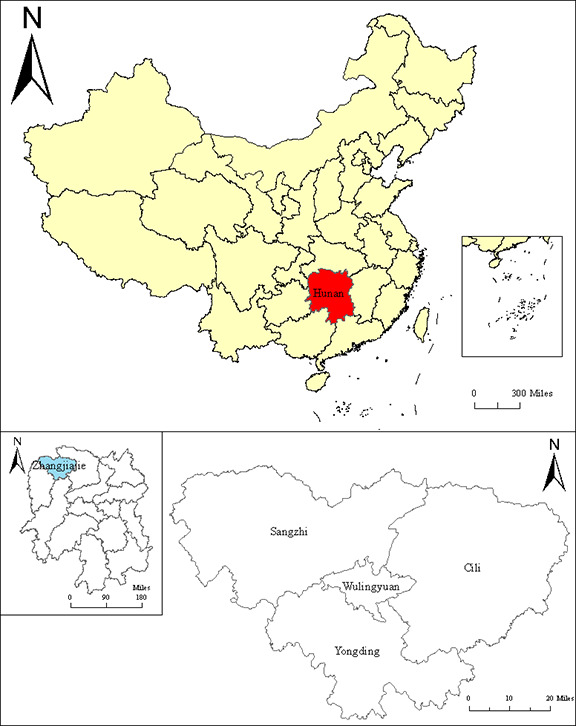
Location of Zhangjiajie City, Hunan Province. The map was obtained from the Standard Map Service website of the Ministry of Natural Resources of China, with the map number GS (2020) 4619 (https://www.resdc.cn/DOI/DOI.aspx?DOIID=129). The map boundaries remained unchanged.

**Fig 2:**
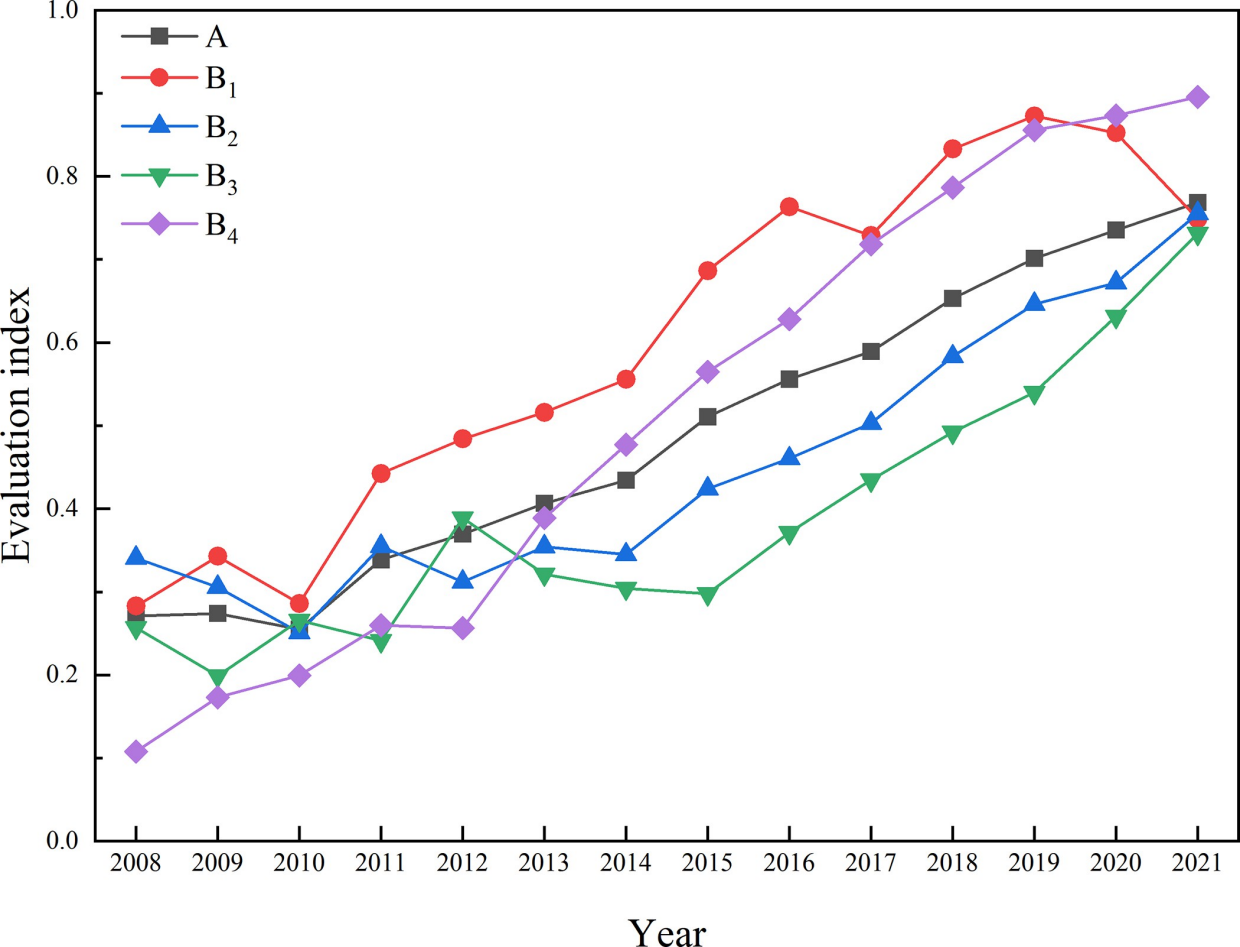
The annual trends in the ecological livability construction level of Zhangjiajie City and the proximity of various systems. A refers to the Ecological livability construction level. B1, B2, B3, and B4 represent the approach degree values of the primary indicators—Ecological development level, Public services and security, Residential life and cultural environment, and Economic development level, respectively, as processed by the entropy-TOPSIS model.

The entropy-TOPSIS model was utilized to indicator calculation, the approach degree values of the ecological livability construction level in Zhangjiajie City and each subsystem, along with the annual trends, are obtained (Table 4 and Fig 2). Over the past 14 years, the ecological livability construction level approach degree in Zhangjiajie City has shown minimal fluctuation and follows a time-evolving trend of initial decline followed by sustained ascent. It decreased from 0.2712 in 2008 to 0.2547 in 2010 and subsequently rose steadily to 0.7686 in 2021, forming a distinctive "J-shaped" pattern characterized by an initial decrease followed by an upward trajectory. The evolution of the ecological livability construction level in different development stages shows significant differences. After different periods of development, it can be roughly divided into a declining period and a downward arc-shaped upward period with a fast-then-slow trend. The approach degree of Zhangjiajie City’s ecological livability construction level with various systems is calculated based on Formula (11). As shown in Table 4 and Fig 2, from 2008 to 2009, the approach degree for public services and security and residential life and cultural environment decreased from 0.3409 and 0.257 to 0.3058 and 0.199, respectively. From 2009 to 2010, the approach degree for ecological development level and public services and security decreased from 0.3428 and 0.3058 to 0.2859 and 0.2512, respectively. During the period of 2008–2010, the ecological livability construction level in Zhangjiajie City was mainly influenced by the systems of residential life and cultural environment, and public services and security, leading to a decline in the approach degree from 0.2712 to 0.2547, entering a declining period. Due to the weak foundation of public services and security in the early stages in Zhangjiajie, the residential life and cultural environment were difficult to sustain. In an effort to improve the challenging situation, there was a focus on rapid economic development, which led to a neglect of environmental pollution control. As a result, the region’s ecological livability construction level gradually declined. In 2010, upon the completion of China’s Eleventh Five-Year Plan, the Zhangjiajie Municipal Party Committee and Municipal Government, in response to the complex challenges posed by the domestic economy, society, and the relationship between humans and nature, proposed the initiative "strengthening the foundation, adjusting the structure, promoting transformation, improving people’s livelihood, and enhancing the overall level" as the main focus to advance the "Five-Year Great Change Project." This led to sustained, steady, and rapid economic development and social harmony throughout the city. Hence, in the periods of 2010–2014, 2014–2017, and 2017–2021, the ecological livability construction level showed three segments of a downward arc-shaped continuous rise with a fast-then-slow trend (Fig 1). By 2021, the approach degree of Zhangjiajie’s ecological livability construction level reached its peak at 0.7686, representing a 183% increase compared to its approach degree in 2010. From an economic theory perspective, the end of the third rising arc indicates the possibility of entering a new upward trend. shaped continuous rise with a fast-then-slow trend. From an economic theory perspective, the end of the third rising arc indicates the possibility of entering a new upward trend.

**Table 4:** The approach degree values of Zhangjiajie’s ecological livability construction level and each system from 2008 to 2021.

Year	Ecological livability construction level	Ecological development level	Public services and security	Residential life and cultural environment	Economic development level
2008	0.2712	0.2829	0.3409	0.257	0.1079
2009	0.2740	0.3428	0.3058	0.199	0.1729
2010	0.2547	0.2859	0.2512	0.2657	0.1993
2011	0.3385	0.4424	0.355	0.2412	0.2596
2012	0.3696	0.484	0.3121	0.3889	0.2564
2013	0.4065	0.5159	0.3544	0.3213	0.3887
2014	0.4344	0.5557	0.345	0.3042	0.4773
2015	0.5105	0.6866	0.4242	0.2977	0.5646
2016	0.5558	0.7634	0.4603	0.3712	0.6282
2017	0.5894	0.7287	0.5029	0.4345	0.7182
2018	0.6532	0.8328	0.5833	0.4921	0.7863
2019	0.7013	0.8727	0.6464	0.5398	0.8556
2020	0.7355	0.8523	0.6721	0.6314	0.8734
2021	0.7686	0.7488	0.7554	0.7312	0.8954

The developmental trends of each subsystem can be divided into two phases. In the early period (2008–2014), due to the implementation of the 12th Five-Year Plan, the coordination of economic development increased, enhancing the resilience of the economic system. However, various aspects such as ecology, social allocation, and residents’ living conditions were immature and unstable. Besides the economic development level, the approach degree values of each subsystem fluctuated significantly and frequently (Fig 2). In the later period (2014–2021), with the coordinated advancement of economic, cultural, and social construction, the balance of development improved, achieving harmonious development among the economy, society, and nature. The approach degree values of each subsystem gradually increased. Among the four major systems contributing to Zhangjiajie City’s ecologically livable construction level, the ecological development level consistently ranked first from 2008 to 2019. With the continuous growth of the economy, the economic development level approach values in 2014, 2017, and 2019 gradually approached the ecological development level, with the differences being 0.0784, 0.0105, and 0.0171 respectively. In 2020, the economic development level approach value surged to 0.8734, surpassing the ecological development level to claim the top spot. The approach of public services and security remained in the first place until 2013 when it stabilized in the third position. Residential life and cultural environment peaked in 2012 and have remained in the fourth position since then. In a comprehensive analysis, the economic development level and ecological development level trends are relatively positive. However, the acceleration of economic growth and an unreasonable industrial structure have to some extent constrained ecological development [65, 66]. Meanwhile, the incomplete social welfare system has impacted the improvement of people’s material living standards, making it challenging for residents to pursue a high-quality life and cultural environment [67, 68]. These two aspects mutually constrain each other, reflecting significant development potential in public services and security, residential life and cultural environment.

### The systemic coupling coordination trend of ecological livability construction level

**Fig 3:**
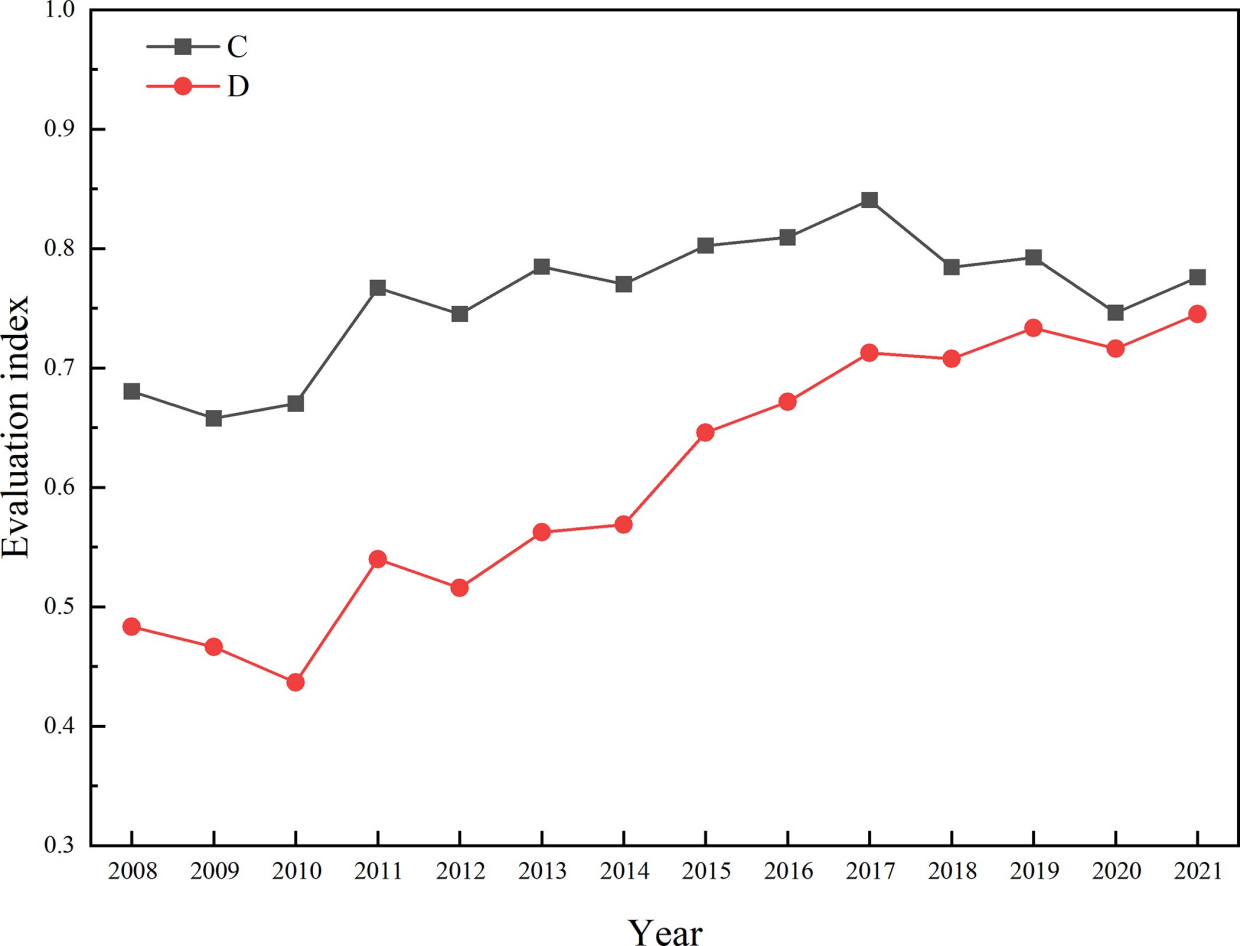
The annual trend of systemic coupling coordination in the ecological livability construction level of Zhangjiajie City.

Coupling degree reflects the interaction and constraint among systems, while coupling coordination degree measures the harmonious consistency and overall synergistic effect among systems, aiding in determining whether the systems are in a state of mutual promotion or constraint on development. The coupling degree (C value) and coupling coordination degree (D value) of Zhangjiajie’s ecological livability system on the basis of Eqs (12)–(14), respectively, are illustrated in Fig 3 and Table 5. Overall, from 2008 to 2021, the coupling degree of Zhangjiajie’s ecological livability construction level systems show an initial increase followed by a decline, while the coupling coordination degree exhibits a slight increasing trend. Regarding the coupling degree, it can be divided into three stages: 2008–2010 is the primary coupling stage, 2011–2014 and 2019–2021 are intermediate coupling stages, and 2015–2017 is the good coupling stage. This trend of initial increase followed by decline, with frequent fluctuations in each stage, indicates an overall intermediate coupling level.

**Table 5:** Coupling degree and coordinated coordination degree of ecological livability construction level systems in Zhangjiajie City from 2008 to 2021.

Year	C value	Coupling level	D value	Coupling coordination level
2008	0.6804	Primary coupling	0.4834	On the verge of imbalance
2009	0.6577	Primary coupling	0.4664	On the verge of imbalance
2010	0.6701	Primary coupling	0.4365	On the verge of imbalance
2011	0.7671	Intermediate coupling	0.5398	Marginally coordinated
2012	0.7452	Intermediate coupling	0.5159	Marginally coordinated
2013	0.7849	Intermediate coupling	0.5626	Marginally coordinated
2014	0.7701	Intermediate coupling	0.5688	Marginally coordinated
2015	0.8025	Good coupling	0.6459	Primary coordinated
2016	0.8093	Good coupling	0.6718	Primary coordinated
2017	0.8406	Good coupling	0.7127	Intermediate coordinated
2018	0.7844	Intermediate coupling	0.7078	Intermediate coordinated
2019	0.7925	Intermediate coupling	0.7334	Intermediate coordinated
2020	0.7463	Intermediate coupling	0.7161	Intermediate coordinated
2021	0.7759	Intermediate coupling	0.7450	Intermediate coordinated

Concerning the coupling coordination degree, it can be categorized into four stages: 2008–2010 is on the verge of imbalance stage, 2011–2014 is the marginally coordinated stage, 2015–2016 is the primary coordinated stage, and 2017–2021 is the intermediate coordination stage. Throughout the entire process, the fluctuation is not significant, showing a slow upward trend (Fig 3). Through this analysis, we find that from 2008 to 2014, the coupling degree among Zhangjiajie’s ecological livability systems fluctuated frequently. During this period, from 2008 to 2010, it was at a primary coupling stage, and the system was on the verge of imbalance. From 2010 to 2014, it entered an intermediate coupling stage, and the coupling coordination. was marginally coordinated. Overall, the coupling degree among ecological livability construction level systems was relatively weak, and the coupling coordination degree was not high. This indicates that during this period, the systems developed relatively independently, and the coupling mechanisms of the four systems were weak, resulting in an uncoordinated state of ecological development level, public services and security, residential life and cultural environment, and economic development level. From 2014 to 2017, the coupling degree and coupling coordination degree gradually increased, entering a good coupling and intermediate coordinated stage. The coupling coordination degree spanned three levels, progressing from marginally coordinated stage to Intermediate coordinated stage. This suggests that the coordinated advancement of economic development, cultural construction, and social development improved the balance of development [69, 70]. Economic, social, human, and natural elements achieved harmonious development and mutual promotion, with all systems moving towards a positive trend. From 2017 to 2021, the coupling level decreased to an intermediate coupling stage, indicating a reduction in the correlation among systems. However, due to the continuous increase in the coupling coordination level, although the mutuality diminished, it also reflects a weakening of the constraining effect among Zhangjiajie’s ecological livability systems.

### Analysis of influencing factors of ecological livability construction level

The degree of obstacle can be obtained by the obstacle diagnosis model (Table 6). The top 5 factors in terms of the degree of obstacle are considered to be the critical obstacle factors. According to the data shown in Table 6, from 2008 to 2021, Zhangjiajie faced several major obstacle factors in its ecological livability construction, with top five major obstacle factors appearing more than 7 times. These are Average wage of in-service employees, the Proportion of social security and employment to fiscal expenditure, per capita postal and telecommunications business volume, per capita public library book collection, and the proportion of education expenditure to fiscal expenditure. This indicates that these factors are the most significant obstacles to the ecological livability construction level. Among the five major obstacle factors, the top three—average wage of in-service employees, the proportion of social security and employment to fiscal expenditure, and per capita postal and telecommunications business volume—are all indicators related to public services and security. The remaining two indicators—per capita public library book collection and the proportion of education expenditure to fiscal expenditure-belong to the residential life and cultural environment category. Zhangjiajie’s economy is predominantly driven by the tourism industry, which has been the primary force behind regional economic development. However, the industrial and infrastructure construction sectors that support economic growth are not well-developed, leading to a limited income base that directly impacts the average wage of in-service employees. Additionally, Zhangjiajie’s location in inland mountainous areas has resulted in a relatively slow urbanization process and limited educational resources. The restrictive topography also diminishes the functionality of transportation hubs, limiting improvements in the quality of life for local residents. Furthermore, the government’s early focus on tourism development led to relatively low investment in libraries, resulting in an insufficient number of books in public library collections, making it difficult to ensure a rich cultural life. This analysis highlights that there is still significant room for improvement in public services and security, as well as in the residential life and cultural environment in Zhangjiajie.

**Table 6:** Top 5 obstacle factors affecting the ecological livability construction level in Zhangjiajie City (2008–2021).

Year	Factor1	obstacle degree	Factor 2	obstacle degree	Factor 3	obstacle degree	Factor 4	obstacle degree	Factor 5	obstacle degree
2008	C16	5.48%	C27	4.88%	C37	4.84%	C24	4.77%	C29	4.76%
2009	C16	5.45%	C27	4.96%	C37	4.86%	C24	4.78%	C29	4.66%
2010	C16	5.45%	C18	4.87%	C37	4.83%	C27	4.77%	C23	4.75%
2011	C29	5.80%	C37	5.44%	C27	5.08%	C23	5.08%	C46	4.90%
2012	C29	6.00%	C23	5.32%	C37	5.00%	C46	4.99%	C27	4.94%
2013	C29	6.39%	C37	5.30%	C14	5.08%	C27	4.90%	C23	4.87%
2014	C29	6.62%	C15	5.53%	C37	5.52%	C23	4.84%	C27	4.67%
2015	C29	7.79%	C15	6.50%	C37	6.25%	C38	5.49%	C28	5.18%
2016	C29	8.06%	C28	7.07%	C38	5.98%	C36	5.51%	C37	5.19%
2017	C29	9.40%	C38	7.80%	C14	6.55%	C28	6.41%	C36	5.46%
2018	C38	10.31%	C29	9.26%	C28	9.20%	C36	6.00%	C31	5.95%
2019	C38	11.62%	C28	9.94%	C31	8.57%	C29	7.32%	C26	6.28%
2020	C38	14.79%	C28	14.68%	C25	9.32%	C13	7.46%	C31	6.01%
2021	C14	19.89%	C38	19.43%	C28	17.53%	C25	10.35%	C31	7.34%

Analyzing these factors from the perspective of various systems contributing to Zhangjiajie’s ecological livability construction level, the Major obstacle factors fall into four categories: (i) Ecological development level: this category includes forest coverage rate and green coverage rate of built-up area. It indicates that vegetation coverage and urban greening significantly impact livable environments; (ii) Public services and security: factors like per capita postal and telecommunications business volume, Average wage of in-service employees, and the proportion of social security and employment to fiscal expenditure highlight employment and social security as major obstacles. Strengthening public services and security measures is crucial; (iii) Residential life and cultural environment: the obstacle factors include per capita public library book collection, the proportion of education expenditure to fiscal expenditure, and population density. This reflects the influence of cultural environment and education levels on ecological livability, emphasizing the need to advance residential cultural environment construction; (iv) Economic development level: factors related to urban-rural income ratio show the economic disparity between urban and rural areas, impacting comprehensive ecological livability development. Overall, the analysis suggests that local employment and livelihood issues pose significant bottlenecks to improving Zhangjiajie’s ecological livability construction level. Efforts to strengthen public services and security are urgently needed. Simultaneously, promoting residential cultural environment construction is essential. As mobile communication technology advances and smartphones become ubiquitous, telecommunications become an integral part of residents’ lives, reflecting the demand for high-quality living conditions. In the construction of Zhangjiajie as a modern international city for ecological livability, attention must be given not only to hardware facilities but also to enhancing educational technology and other soft infrastructure. Education can directly contribute to an increase in people’s sense of well-being, and the improvement of population quality also benefits ecological conservation.

Examining the obstacle factor rankings for each year, from 2008 to 2009, the main obstacles were forest coverage rate, number of hospital beds per 10,000 people, Average wage of in-service employees, per capita postal and telecommunications business volume, and per capita public library book collection. This is related to the national emphasis on infrastructure development, particularly in communication and healthcare reforms. However, due to the relatively low forest coverage rate of 68.67%, its impact on the environment is significant. After 2010, number of doctors per 10,000 people and hazard-free treatment rate of domestic garbage have become significant major obstacles. This is attributed to the increasing national investment in social infrastructure. The increase in grassroots medical positions is fundamental to the development of the health sector. The expansion of medical positions plays a crucial role in addressing the overall weakness and structural imbalances in the health sector, contributing to the improvement of medical facilities in urban development. Additionally, with the continuous development of urbanization, the amount of urban waste generation has been increasing. Finding ways to achieve waste reduction and harmlessness in urban areas is essential for environmental protection. During the period of 2011–2014, China entered a crucial phase in the 12th Five-Year Plan. Recognizing the need for strategic adjustments in line with the Four-Pronged Comprehensive Strategy, the Chinese government proactively adapted to and steered the economy toward the new normal. Significantly prioritizing employment, the government-initiated reforms to foster mass entrepreneurship and innovation. This strategic focus injected new vitality into the national economy, resulting in a more intricate industrial structure. Consequently, amidst a backdrop of slowed economic growth, total employment not only remained stable but even exhibited an upward trend. Simultaneously, increased employment opportunities in rural areas led to higher incomes, contributing to a narrowing urban-rural income gap. The urban-rural income ratio decreased from 3.463 in 2010 to 3.419 in 2012, emerging as a newly prominent obstacle factor.

During this period, the continuous development of the urban economy expanded people’s expectations beyond basic living needs and material conditions. People began to seek the tranquility, greenery, and harmony brought by urban greenery, resulting in an increase in the obstacle degree of the green coverage rate of built-up area. However, due to the ongoing development of the industrial economy, the issue of economic growth at the expense of the environment gradually became more pronounced [66, 71]. In terms of specific indicators, the rate of good air quality was only 71.1% in 2014, and the increase in obstacle factors related to the rate of good air quality added to the degree of obstacle, becoming a primary obstacle factor limiting the improvement of Zhangjiajie’s ecological livability level. In 2015, a crucial year for education reform, the Chinese government successively introduced key directions and focused measures for comprehensive reform in the education sector. The policies aimed at promoting fair development and quality improvement in education. Under the influence of these policies, from 2015 to 2018, the proportion of education expenditure to fiscal expenditure fluctuated between 5.49% and 10.31%. The basic education student-teacher ratio became a primary obstacle factor during this period. However, due to economic development and changes in the industrial structure, the increase in market personnel, inadequate growth in employment opportunities, and a rise in unemployment contributed to the increased obstacle degree of social security and employment expenditure to fiscal expenditure. In 2019–2021, green coverage rate of built-up area and proportion of education expenditure to fiscal expenditure continued to rise. The contribution of these factors to Zhangjiajie’s ecological livability construction increased, emphasizing the focus on education, urban living environments, and employment issues in recent years.

## Conclusions and suggestions

### Conclusions

This study constructed an index system for the ecological livability construction level in Zhangjiajie City from 2008 to 2019 under the synergistic perspective. The entropy-TOPSIS model was employed to evaluate the ecological livability construction level. The coupling coordination degree model was used to explore the correlation and coordination among systems. Additionally, the obstacle diagnosis model was introduced to investigate the main obstacle factors for improving the ecological livability construction level. The conclusions are as follows:

First, the ecological livability construction level in Zhangjiajie City has shown a trend of first decline and then continuous rise, with the approach degree increasing from 0.2712 to 0.7686. The evolution of healthy city construction levels varies in different stages, broadly categorized into the declining period, 2010–2014, 2014–2017, and 2017–2021. There are three segments of downward arcs followed by slow upward trends, and a new rising trend is anticipated. The development trends of each system can be divided into two stages: in the early period (2008–2014), the approach degree values of each system fluctuated significantly, while in the later period (2014–2021), the approach degree values of each system gradually increased. Public services and security, as well as residential life and cultural environment, have shown relatively lagging.

Second, from 2008 to 2021, the coupling degree of the four major ecological livability systems in Zhangjiajie City showed an initial increase followed by a subsequent decline, while the coupling coordination degree exhibited a slow upward trend. In terms of coupling degree, there was an overall trend of first increasing and then decreasing, with frequent fluctuations in each stage, resulting in a moderate coupling level overall. The coupling coordination degree showed minimal fluctuation and a slow upward trend. In the period from 2008 to 2010, the coupling degree was at a primary coupling and on the verge of imbalance stage. From 2010 to 2014, it reached an intermediate level, indicating a barely coordinated state. From 2014 to 2017, the coupling degree transitioned to a good level, preliminarily entering an intermediate coordination stage. The coupling coordination level spanned three stages, evolving from a barely coordinated state to an intermediate coordination level. From 2017 to 2021, the coupling level dropped to an intermediate level, reflecting a weakening correlation among systems. However, due to the continuous improvement in coupling coordination level, it also indicated a reduction in the constraining effect among the ecological livability systems in Zhangjiajie City.

Third, from the analysis of obstacle factors, the average wage of in-service employees, the proportion of social security and employment to fiscal expenditure, per capita postal and telecommunications business volume, per capita public library book collection, and the proportion of education expenditure to fiscal expenditure have become the primary influencing factors on the ecological livability construction level in Zhangjiajie. From the first obstacle factor to the fifth obstacle factor, the degree of aggregation gradually diminishes. The primary obstacle factors can be classified into four categories based on system type. Ecological development level category: forest coverage rate and green coverage rate of built-up area; Public services and security category: per capita postal and telecommunications business volume, Average wage of in-service employees, proportion of social security and employment to fiscal expenditure; Residential life and cultural environment: per capita public library book collection, proportion of education expenditure to fiscal expenditure, population density; Economic development level category: urban-rural income ratio. From 2008 to 2021, the main obstacle factors in Zhangjiajie’s ecological livability construction level, which ranked in the top 5, had five obstacle factors with frequencies exceeding 7 times. They are, respectively: average wage of in-service employees, proportion of social security and employment expenditure to fiscal expenditure, per capita postal and telecommunications business volume, per capita public library book collection, and proportion of education expenditure to fiscal expenditure.

### Suggestions

After reaching its lowest point in 2010, the ecological livability construction level in Zhangjiajie has shown a generally steady increase. In the periods of 2010–2014, 2014–2017, and 2017–2021, there have been three phases of continuous upward trends resembling a downward curve followed by a slow rise. Explaining this through economic theory, the ends of the third rising curve indicates the onset of a new upward trend. The decline in coupling level to intermediate coupling reflects a weakening correlation among various systems. However, due to the continuous improvement in coupling coordination level, despite the reduction in mutual influence, it also indicates a diminishing restrictive effect among the ecological livability systems in Zhangjiajie. This, in turn, suggests that ecological, economic, social, and livelihood aspects are progressing positively and harmoniously, moving toward a direction of active and harmonious development. Zhangjiajie should learn from past development experiences and seize the opportunities presented by the new upward trend. Based on this, the following recommendations are proposed in this study:

First, strictly regulate environmental pollution and promote ecological sustainability. Zhangjiajie’s level of industrialization is relatively low, leading to minimal ecological damage. As a result, the ecological development level poses a relatively small obstacle to improving the city’s ecological livability construction level, creating favorable conditions for the development of an ecologically livable area. However, several concerning trends have emerged, such as the increase in acid rain in 2014, with the rate of good air quality dropping to just 71.1%, and from 2019 to 2021, the obstacle degree related to the green coverage rate of the built-up area reached 19.89%. These phenomena indicate that, although Zhangjiajie’s overall ecological environment is relatively good, economic development has caused some ecological degradation. Therefore, attention to ecological protection should keep pace with economic growth. Zhangjiajie should fully leverage its unique geographical location and natural wonders by actively advancing karst area management, protecting natural forest resources, and improving key projects such as agricultural and rural public infrastructure, urban sewage and waste treatment facilities, resource conservation, and pollution control. Additionally, the city should increase investment in energy conservation and environmental protection to ensure sustainable development.

Second, optimizing government resource allocation to reduce the wealth gap. The primary obstacle factor under the Economic development level system is the urban-rural income ratio. The key to enhancing the economic development level in Zhangjiajie is to reduce wealth disparities and narrow the urban-rural gap. There is a significant disparity in the development levels between urban and rural areas, with clear hierarchical phenomena. It is essential to optimize resource allocation, increase employment opportunities, conduct public activities such as employment guidance lectures, combat fraudulent behaviors that exploit job seekers’ psychological vulnerabilities, and create a favorable employment environment. Promoting equitable development and improving the quality of education are crucial. This involves increasing fiscal expenditures to ensure residents’ livelihoods, reducing redundant institutional structures, improving industrial institutions, and actively developing rural characteristic economies such as agricultural landscape tourism, farming experiences, and rural leisure tourism to reduce wealth disparities between urban and rural areas.

Third, improve the social security system and increase employee income levels. Public services and security ranks third in the ecological livability construction level. The primary issues include a low average wage of in-service employees, a relatively low Proportion of social security and employment to fiscal expenditure, among other prominent problems. These factors significantly affect the public services and guarantee level in Zhangjiajie. To address this, the Zhangjiajie Municipal Government can address the issue of low average wages for in-service employees in the region by strengthening macroeconomic regulation of corporate wages, establishing a sound mechanism for regular wage increases, and adjusting the indicators for determining the contribution base of employees’ basic pension insurance. At the same time, it is necessary to improve the social security system, enhance the employment service system, and provide free skills training to help individuals expand their employment opportunities and development paths. Additionally, the government should expedite institutional reforms, eliminate redundant positions, reduce salaries for non-essential roles, and take other measures to alleviate the burden on fiscal expenditure, thereby better supporting social security and infrastructure development.

Fourth, increase cultural and educational expenditures to enhance the cultural atmosphere of the city. The aspect of residential life and cultural environment lags behind, ranking fourth. The main obstacle factors restricting the improvement of residents’ living standards are the proportion of education expenditure to fiscal expenditure and Per capita public library book collection. Regarding education investment, it is essential to ensure that fiscal allocations and per capita fiscal allocations truly achieve an "only increase, not decrease" approach. Implement targeted assistance projects in education, addressing the issue of "urban crowding, rural weakness" to achieve balanced and high-quality compulsory education, ultimately realizing the integration of urban and rural education. Concurrently, efforts should be made to optimize the improvement of regional cultural and educational infrastructure, actively promote cultural undertakings and industry investment, and enhance the cultural atmosphere, thereby elevating the humanistic aspect of Zhangjiajie’s ecological livability construction level.

## Supporting information

S1 Table Raw data for this study. (DOCX)
