# Supplementary material for: Evaluation of urban ecological livability and obstacle factor diagnosis from a synergistic perspective: A case study of Zhangjiajie City, China
Source: PLoS One. 2024 Nov 15;19(11):e0313267. doi: 10.1371/journal.pone.0313267 (PMC11567568; doi:10.1371/journal.pone.0313267)
Supplement: S1 Table — (DOCX) [file pone.0313267.s001.docx]

**S1 Table.** Raw data for this study

| Year | 2021 | 2020 | 2019 | 2018 | 2017 | 2016 | 2015 | 2014 | 2013 | 2012 | 2011 | 2010 | 2009 | 2008 |
| --- | --- | --- | --- | --- | --- | --- | --- | --- | --- | --- | --- | --- | --- | --- |
| (C11) Energy Consumption per Unit of GDP (Ton of standard coal/10,000 RMB) | 0.486213888 | 0.505946 | 0.52197 | 0.543606 | 0.573363 | 0.608214 | 0.608579 | 0.602554 | 0.634 | 0.657 | 0.7 | 0.859 | 0.9006 | 0.92 |
| (C12) Electricity consumption per unit of GDP (kW·h//10,000 RMB) | 536.8076943 | 520.667 | 531.6726 | 539.0031 | 534.4072 | 542.3251 | 512.837 | 497.9 | 546 | 533.4 | 549.7 | 659.2 | 681.2895 | 648 |
| (C13) Water consumption per unit of GDP (m3/10,000 RMB) | 1.080194386 | 1.383572 | 1.200016 | 0.807844 | 0.797436 | 0.799027 | 0.945765 | 1.055858 | 1.248002 | 1.316767 | 1.943216 | 1.556957 | 1.6958 | 1.807767 |
| (C14) Green coverage rate of built-up area (%) | 34.75 | 41.66 | 41.84 | 38.06 | 35.02 | 39.47 | 39.5 | 35.47 | 33.59 | 33.4 | 37.72 | 36.45 | 38.64 | 38.76 |
| (C15) rate of good air quality (%) | 97.8 | 98.1 | 93.7 | 93.2 | 88.8 | 81.9 | 71.27 | 71.1 | 74.5 | 92.1 | 92 | 91.2 | 94.8 | 91 |
| (C16) Forest coverage rate (%) | 71 | 71 | 70.99 | 70.99 | 70.98 | 70.98 | 70.98 | 69.62 | 69.62 | 69.6 | 69.6 | 68.75 | 68.75 | 68.67 |
| (C17) Treatment rate of domestic sewage (%) | 96.7 | 96.7 | 96.7 | 95.22 | 87.12 | 84.32 | 81.09 | 80.74 | 78.27 | 56.98 | 61.97 | 61.97 | 60 | 44.75 |
| (C18) Hazzard-free treatment rate of domestic garbage | 100 | 100 | 100 | 100 | 100 | 100 | 100 | 100 | 100 | 80 | 67 | 51.73 | 65 | 51.99 |
| (C21) Natural gas coverage rate (%) | 92.78 | 91.9 | 91.5 | 91.5 | 91.1 | 90.92 | 90.31 | 86.49 | 82.3 | 76.71 | 72.28 | 55.79 | 69.15 | 68.71 |
| (C22) Highway miles per 10,000 people (km/10,000 people) | 61.18651923 | 60.67756 | 58.46243 | 58.73594 | 58.8796 | 58.44614 | 58.26772 | 58.47926 | 58.56916 | 58.39784 | 58.65157 | 58.38319 | 45.64022 | 43.81042 |
| (C23) Number of doctors per 10,000 people (ten thousand person) | 27.33893928 | 26.5444 | 27.89512 | 21.86983 | 20.486 | 21.02231 | 19.612 | 18.13547 | 17.53294 | 15.75846 | 15.81805 | 14.79176 | 16.39409 | 16.39409 |
| (C24) Number of hospital beds per 10,000 people (ten thousand person) | 71.27722969 | 68.86711 | 66.457 | 71.76058 | 63.75555 | 55.9955 | 52.73125 | 48.27518 | 47.16282 | 42.44852 | 39.27696 | 37.0721 | 34.0727 | 33.03317 |
| (C25) Urban registered unemployment rate (%) | 2.99 | 3.6 | 2.9 | 2.05 | 3.76 | 3.8 | 3.29 | 3.45 | 3 | 3 | 1.18 | 4.3 | 4.43 | 2 |
| (C26) The ratio of disposable income to house price of urban residents | 0.194052384 | 0.207449 | 0.237363 | 0.228274 | 0.192569 | 0.189024 | 0.187125 | 0.254073 | 0.206856 | 0.203945 | 0.203187 | 0.203887 | 0.173904 | 0.18124 |
| (C27) Average wage of in-service employees (RMB) | 82221 | 79586 | 70521 | 70633 | 63884 | 58259 | 48425 | 42332 | 38131 | 34454 | 31080 | 26493 | 24311 | 23581 |
| (C28) Proportion of social security and employment to fiscal expenditure (%) | 13.36685065 | 12.13622 | 12.99814 | 12.33843 | 13.02086 | 11.77523 | 12.96646 | 13.41412 | 14.22623 | 14.48645 | 15.67388 | 15.06987 | 17.78859 | 18.43146 |
| (C29) Per capita postal and telecommunications business volume (RMB/people) | 11993.64365 | 9006.723 | 6666.667 | 3833.799 | 1442.935 | 1896.54 | 1057.087 | 1012.508 | 816.7548 | 723.6536 | 656.3318 | 1747.06 | 1431.886 | 862.6796 |
| (C31) Population density (per people/km2) | 158.4286164 | 159.1524 | 162.5092 | 161.3238 | 160.663 | 160.4007 | 159.8657 | 159.3412 | 158.6489 | 157.5684 | 156.3097 | 155.0509 | 158.2398 | 156.981 |
| (C32) Natural population growth rate (%) | -0.93 | 2.05 | 2.9 | 3.89 | 5.39 | 4.22 | 4.55 | 4.79 | 6.21 | 6.33 | 6.14 | 6.87 | 7.98 | 7.32 |
| (C33) Per capita savings deposit balance | 34717.67199 | 30693.18 | 27449.34 | 29894.16 | 25349.17 | 22797.84 | 21011.99 | 20189.92 | 17470.4 | 15038.36 | 12431.41 | 9980.042 | 8070.056 | 6631.794 |
| (C34) Number of household cars per hundred households | 34.6287327 | 31.70015 | 28.65493 | 25.66506 | 22.06547 | 18.57732 | 15.00468 | 11.6694 | 10.02676 | 8.809316 | 7.195681 | 6.941941 | 4.292771 | 2.911196 |
| (C35) University students per 10,000 people (ten thousand people) | 140.1244786 | 121.4606 | 104.5959 | 96.91788 | 90.20632 | 86.42993 | 82.72966 | 79.49309 | 78.93414 | 80.82019 | 85.8533 | 85.03484 | 78.89957 | 71.31306 |
| (C36) Basic education student-teacher ratio | 6.642094645 | 6.340702 | 6.176224 | 5.926169 | 5.810746 | 5.707071 | 5.734229 | 5.969621 | 5.884671 | 5.973319 | 5.953862 | 6.139963 | 6.133667 | 6.045372 |
| (C37) Per capita public library book collection | 3351.65199 | 2915.898 | 2787.245 | 2613.954 | 2533.299 | 2001.177 | 1528.871 | 1448.321 | 1421.582 | 1397.643 | 1140.863 | 1073.811 | 1060.656 | 1002.339 |
| (C38) Proportion of education expenditure to fiscal expenditure (%) | 0.149024537 | 0.139551 | 0.139452 | 0.133291 | 0.13645 | 0.148765 | 0.146705 | 0.162932 | 0.189287 | 0.221801 | 0.13885 | 0.136141 | 0.152253 | 0.182058 |
| (C41) Per capita GDP (RMB) | 38333 | 36708 | 35767 | 37719 | 35170 | 32300 | 29425 | 27051 | 24259 | 22658 | 20082 | 16238 | 13517 | 12337 |
| (C42) Proportion of tertiary industry to GDP (%) | 71.65555153 | 69.57139 | 72.68249 | 72.1 | 68.5 | 67.2 | 65.6 | 63.7 | 62.5 | 48.7 | 61.1 | 64.5 | 65.6 | 59.6 |
| (C43) Per capita retail sales of social consumer goods (RMB) | 13560.21982 | 12984.45 | 16369.74 | 14961.96 | 14311.83 | 12948.79 | 11640.42 | 9111.257 | 7452.394 | 6515.545 | 6568.016 | 5618.023 | 4637.72 | 3935.85 |
| (C44) Per capita fixed asset investment (RMB) | 23432.09584 | 29156.91 | 26938.35 | 25172.81 | 22936.8 | 19992.15 | 19448.82 | 16517.45 | 13938.11 | 11324.15 | 9744.312 | 8387.795 | 6430.892 | 1984.631 |
| (C45) The urbanization rate (%) | 52.37 | 51.65 | 50.48 | 49.2 | 48.02 | 46.06 | 44.61 | 43.32 | 42.23 | 41.1 | 39.15 | 36.19 | 38.6 | 37.1 |
| (C46) Urban-rural income ratio | 2.350619623 | 2.416706 | 2.558355 | 2.596246 | 2.639669 | 2.695463 | 2.744996 | 2.85139 | 2.924678 | 3.419373 | 3.444098 | 3.463438 | 3.514652 | 3.587296 |
| (C47) Proportion of added value of high-tech industries to GDP (%) | 4.81138741 | 4.153194 | 3.901467 | 3.086627 | 2.608836 | 1.933928 | 1.685347 | 1.73611 | 1.003446 | 1.252279 | 2.580191 | 2.244804 | 1.997277 | 1.831938 |
| (C48) Labor productivity (RMB/people) | 11555.00442 | 11143.21 | 9424.495 | 8797.072 | 8104.114 | 7772.833 | 7423.505 | 6756.239 | 6091.829 | 5569.077 | 4976.355 | 4309.349 | 3494.449 | 2826.141 |
